# Reply

**Published:** 2010

**Authors:** Xiaofang Gao

**Affiliations:** Department of Respiratory Medicine, Chinese People’s Liberation Army General Hospital, 28 Fuxing Road, Beijing, 100853, China T: +86-15010682841 fangzhai@tom.com

We appreciate the comments of Dr. Wiwanitkit on our article. All procedures for the serological test in this study were performed according to the *Guideline for Internal Quality Control for Quantitative Measurements in Clinical Laboratories of China*. The primary aim of this pilot study was to investigate the approximate incidence of invasive pulmonary aspergillosis in patients with acute exacerbation of COPD and the diagnostic value of combined serological tests, so further analysis on cost-effectiveness and diagnostic protocol is limited by the relatively small number of confirmed cases. We think that the possible solution to this problem is large multicenter clinical trial conducted in high-risk population.

